# A PDMS Coating-Based Balloon-Shaped Fiber Optic Respiratory Monitoring Sensor

**DOI:** 10.3390/s25165174

**Published:** 2025-08-20

**Authors:** Qingfeng Shi, Yunkun Cui, Yu Zhang, Jie Zhang, Feng Peng

**Affiliations:** 1 Sports Department, Taishan University, Taian 271000, China; tsxy0538tiyu@163.com; 2 School of Physics and Technology, University of Jinan, Jinan 250022, China; zhangyu456729@163.com (Y.Z.); zhangjie1102025@163.com (J.Z.)

**Keywords:** PDMS, balloon shape, Mach–Zehnder interferometer, temperature, respiratory monitoring sensor

## Abstract

A respiratory monitoring sensor based on a balloon-shaped optical fiber is proposed. The sensor consists of a single-mode fiber (SMF) coated with polydimethylsiloxane (PDMS) bent into a balloon shape to form a fiber optic Mach–Zehnder interferometer. The sensor’s sensitivity to temperature enables monitoring of breathing status by recognizing the temperature changes that occur during human respiration. By adjusting the bending radius of the balloon-shaped SMF, high-order modes can be effectively excited to interfere with the core mode. Due to the high thermo-optic coefficient and thermal expansion coefficient of PDMS itself, the balloon-shaped fiber optic sensor can achieve temperature sensitivity. The experimental results show that the temperature sensitivity is −166.29 pm/°C in a temperature range of 30 °C to 60 °C. Finally, the proposed sensor was mounted into a respiratory mask to monitor different breathing states (normal, fast, slow, and oral–nasal breathing transitions) and breathing frequencies.

## 1. Introduction

As the most basic physiological activity for sustaining life, real-time monitoring of respiratory status is one of the key parameters for assessing an individual’s basic physical features and overall health level. In health monitoring, respiration monitoring is a key method for assessing physical health, as well as a significant means of evaluating cardiorespiratory endurance. In competitive sports, respiratory monitoring is widely used throughout the entire process, from athlete selection to training and competition. Through respiratory monitoring, the cardiopulmonary potential and recovery ability of athletes can be assessed. Moreover, real-time monitoring of an athlete’s respiratory state can enhance their respiratory efficiency, accurately regulate exercise load, and prevent sports injuries.

In recent years, most wearable respiratory monitoring instruments have utilized electronic sensors, such as piezoelectric film [1,2] and flexible capacitive electrodes [3,4,5]. Although these sensors can achieve accurate and real-time respiratory monitoring, they still have some shortcomings. For example, the poor biocompatibility of certain electronic metal components can pose long-term health hazards. Additionally, these sensors are susceptible to electromagnetic field disruptions and electrochemical erosion. Fiber optic sensors offer significant advantages in wearable respiratory monitoring due to their corrosion resistance, immunity to electromagnetic interference, and high sensitivity. Furthermore, fiber optic sensors are easily networked and transmit data over long distances, which is of great significance for medical networks and telemedicine [6,7]. Various sensors based on fiber Bragg gratings (FBGs) [8,9], specialty optical fibers [10,11,12], and fiber optic interferometers [7,13,14] have been developed in the field of wearable breathing sensors. Wang et al. offset-spliced few-mode fiber with a single-mode fiber (SMF) to form a Mach–Zehnder interferometer (MZI), achieving 8.53 dB/m^−1^ in a curvature range of 2.7 m^−1^ to 4.1 m^−1^, and encapsulated it in a belt to monitor human respiration [15]. Pang et al. embedded a 30 mm multimode fiber into an SMF to form an MZI, achieving −5.76 dB/m^−1^ in a curvature range of 2.2535 m^−1^ to 3.1722 m^−1^, and then encapsulated it in an elastic waistband to enable the collection and analysis of respiratory signals [16]. However, body movement affects the strain signal, thereby reducing measurement accuracy. Since the respiratory process causes rapid variations in relative humidity, it is possible to monitor respiratory patterns by monitoring variations in relative humidity. Li et al. side-polished SMF and then deposited MoS_2_ on its surface, achieving a humidity response time of 0.85 s and the monitoring of respiration status [17]. Lai et al. proposed an eccentric fiber Bragg grating (EFBG), which allowed the sensor to directly sense humidity fluctuations during respiration without incorporating any sensitizing material, achieving a response time of 92 ms [9]. Guo et al. coated a layer of LiBr-doped silk fibroin (SF) film on a surface plasmon resonance (SPR) sensor based on plastic-coated optical fibers, which achieved a humidity sensitivity and response time of −6.5 nm/%RH and 135 ms, respectively, enabling the monitoring of respiration status [12]. Qian et al. proposed a fiber optic respiration monitoring sensor based on a micro-tapered long-period fiber grating structure, achieving a humidity sensitivity of 0.048 dB/%RH and a response time of 237 ms, accomplishing the monitoring of different respiration frequencies [18]. Temperature variations can also be used to monitor the respiration pattern. However, the low thermal–optical coefficient of quartz fiber itself is insufficient for detecting the slight temperature variations that occur during respiration. Polydimethylsiloxane (PDMS) can be used to improve the sensitivity of fiber optic temperature sensors due to its high stability, high thermal–optical coefficient [19], low cost, and high biocompatibility. Li et al. proposed an SMF–hollow-core fiber (HCF)–SMF structure, which was side-polished and then filled with PDMS, achieving a temperature sensitivity of 4.223 nm/°C and an ultra-fast response time of 16 ms and enabling the recognition of different breathing modes [20]. These sensors, however, require the use of expensive equipment and complex fabrication methods, increasing the associated costs. Furthermore, structural processing on optical fibers tends to reduce the robustness of the sensors. Therefore, developing a cost-effective and straightforward fiber optic sensor structure for wearable respiratory monitoring sensors is necessary.

In this paper, a balloon-shaped fiber optic respiratory monitoring sensor with PDMS coating is proposed. The sensor consists of an SMF coated with PDMS and bent into a balloon-shaped structure, forming an MZI. When the temperature around the sensor changes, it causes a change in the phase of the higher-order modes transmitted in the balloon-shaped SMF, ultimately leading to shifts in the interference dips. Experimental results show that the temperature sensitivity of the sensor is −166.29 pm/°C within the range of 30–60 °C. Then the sensors are encapsulated into the mask to enable measurement of different breathing states and breathing frequencies. The sensor avoids complex fiber optic fabrication processes, significantly improving fabrication feasibility and cost-effectiveness while ensuring the durability of the sensor. This has broad application prospects in the fields of public health management and competitive sports.

## 2. Theory and Sensing Principle

**Figure 1:**
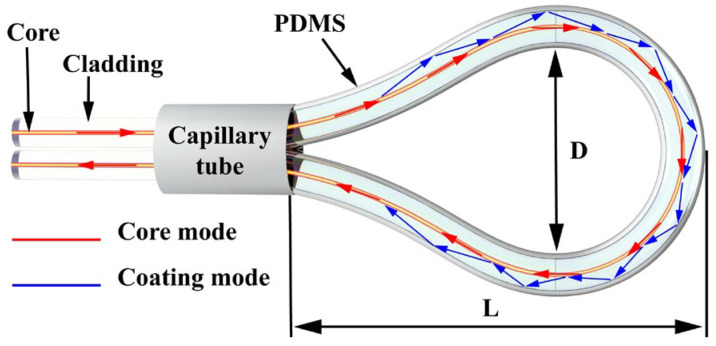
Schematic structure of a balloon-shaped fiber coated with PDMS.

Figure 1 shows a schematic diagram of the MZI of a balloon-shaped SMF coated with PDMS. The bending radius and cross-sectional length of the balloon shape are defined as R and L. When incident light propagates along the core of an SMF and enters a bent SMF, part of the light leaks from the core into the cladding. Since the refractive index of PDMS after curing is 1.41, which is smaller than the refractive index of the cladding, and the balloon-shaped fiber has a large bending radius, total reflection occurs out of the cladding-external medium boundary to form a whispering gallery mode (WGM) [21]. After the excited WGM leaves the bent SMF, part of the WGM will be re-coupled to the fiber core. In addition, since the difference in effective refractive index (RI) and optical path length experienced by the optical signals transmitted in the WGM and the core mode, MZI is formed, and the transmitted intensity of the interferometer can be expressed as [22]

(1) $$I=I_{\mathrm{core}}+I_{WGM}+2\sqrt{I_{\mathrm{core}}I_{\mathrm{WGM}}}\cos\left(\varphi_{0}+\varphi \right)$$

where $I_{\mathrm{core}}$ and $I_{WGM}$ are the strengths of the core mode and WGM, respectively. $\varphi_{0}$ is the initial phase and φ is the phase difference between the core mode and the WGM, expressed as

(2) $$\varphi =\frac{2\pi L_{eff}}{\lambda }\Delta n_{eff}$$

where λ is the free space wavelength, $\Delta n_{eff}$ is the effective refractive index difference between the core mode and WGM, and $L_{eff}$ is the effective bending length. When φ satisfies the destructive interference ($\varphi =\left(2m+1\right)\pi ,m=0,1,2\ldots$), the wavelength position of the interference dips can be defined as

(3) $$\lambda_{m}=\frac{2L_{eff}\Delta n_{eff}}{2m+1}$$

When the temperature around the sensor changes, the thermo-optic coefficient (TOC) and thermal expansion coefficient (TEC) of PDMS itself cause changes in $\Delta n_{eff}$ and $L_{eff}$. Therefore, the wavelength shift of the sensor can be expressed as [23]

(4) $$\frac{\Delta \lambda }{\lambda }=\left(\alpha +\xi \right)\Delta T$$

where α and ξ are the TOC and TEC of the material, respectively, and the PDMS coating improves the temperature sensitivity because it has higher TOC and TEC than quartz fiber.

## 3. Sensor Fabrication and Experimental Results

### 3.1. Experimental Platform Construction

**Figure 2:**
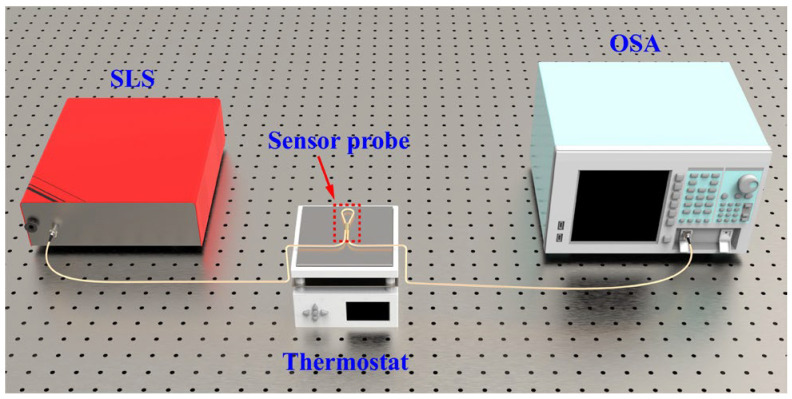
Schematic diagram of the temperature measurement experimental platform.

An experimental setup for temperature measurement was built, as shown in Figure 2. The experimental system utilizes a supercontinuum light source (SLS, NKT Photonics, Copenhagen, Denmark, Superk COMPACT, 450–2400 nm) as the experimental light source and an optical spectrum analyzer (OSA, YOKOGAWA, Tokyo, Japan, AQ6370D, 600–1700 nm) to record the transmission spectrum of the sensor. The thermostat (Bakon, Shenzhen, China, BK946S, temperature controlling range 20–350 °C) was used to adjust the temperature of the sensor’s surrounding environment.

### 3.2. Sensor Design

#### 3.2.1. Numerical Simulation

**Figure 3:**
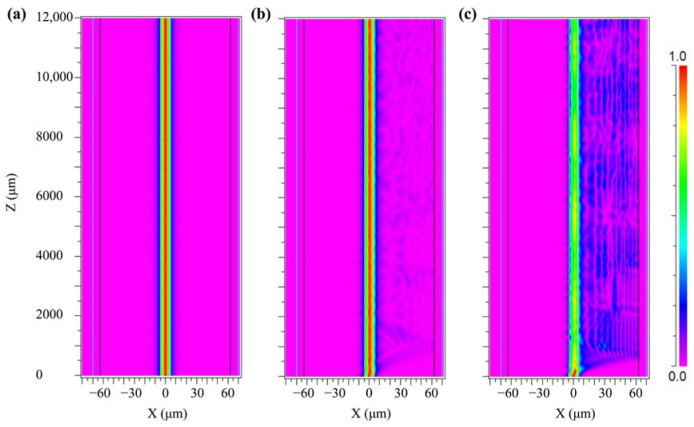
Simulated distribution of the optical field of a balloon-shaped fiber structure at different bending radii. (**a**) Unbent SMF. (**b**) SMF with a bending radius of 10 mm. (**c**) SMF with a bending radius of 4 mm.

To study the light field distribution in a bent fiber, numerical simulations were conducted using the beam propagation method (BPM). Since the top structure of the balloon-shaped optical fiber has a uniform bending radius, only this part was selected for numerical simulation to simplify the simulation process. Moreover, the bent SMF section causes asymmetry in the refractive index distribution due to the elastic–optical effect, the bent SMF can be equated to a straight SMF using conformal mapping. The diameter and refractive index of the core/cladding of the SMF are 8.2/125 μm and 1.4504/1.4447, respectively. The thickness and refractive index of the PDMS are set to 15 μm and 1.41, respectively. The simulated lengths of the SMF and the PDMS are 1.2 cm, and the incident wavelengths are set to 1550 nm. When the fiber is not bent, and the bending radii are 10 mm and 4 mm, the simulation results are shown in Figure 3. It can be seen that the optical field in the unbent fiber is mainly concentrated in the core, and when the fiber is bent, the optical field is mainly concentrated outside the bent fiber. As the bending radius decreases from 10 mm to 4 mm, the intensity of the light field leaking from the core to the cladding increases. Due to the different distributions of light field intensity in the core and cladding, different interference effects are produced, resulting in different extinction ratios (ERs). Therefore, a suitable bending radius needs to be experimentally determined to obtain an effective interference pattern.

#### 3.2.2. Fabrication

The SMF used in the experiments was Corning SMF-28. (Corning Incorporated, New York, NY, USA). Firstly, the coating layer of the SMF was removed over a 2 cm area and wiped with alcohol. Then, the SMF was placed horizontally on the lifter and secured with the clamp. The motor of the lifter was controlled to immerse the SMF in PDMS, using a 10:1 ratio of elastomeric polymer (Sylgard 184-A, Merck KGaA, Darmstadt, Germany) and curing agent (Sylgard 184-B, Merck KGaA, Darmstadt, Germany) at a speed of 500 μm/s. The SMF was placed in the PDMS for 30 s and then in the PDMS for 30 s at a speed of 500 μm/s. The SMF was left in the PDMS for 30 s before being lifted out at 500 μm/s and placed on a heating table for 3 h at a temperature of 80 °C for heat curing. Finally, the SMFs were bent to a suitable bending radius and encapsulated using a capillary tube and UV glue.

**Figure 4:**
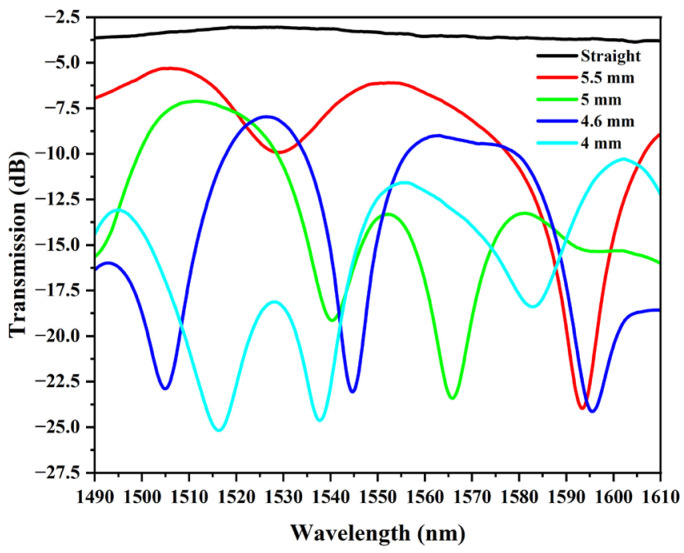
Transmission spectra of PDMS-coated balloon-shaped fiber structures at different bending radii.

To determine a suitable bending radius, the PDMS-coated SMF was bent, and the transmission spectra for different bending radii were recorded. The results are shown in Figure 4. For bending radii larger than 5.5 mm, there is no obvious interference in the wavelength range because there is little optical energy coupled to the cladding and coating in the fiber core. With a bending radius of 5.5 mm, the transmission spectrum exhibits one obvious interference dip. At a bending radius of 5 mm, multiple interference dips gradually appear in the transmission spectrum. Further reducing the bending radius to 4.6 mm resulted in three interference dips with large ERs in the transmission spectrum, while obtaining a suitable free spectral range (FSR). With the bending radius reduced to 4 mm, both the FSR and ER of the interference dips of the transmission spectra started to decrease, which was attributed to the fact that the interference effect of the MZI started to weaken as a result of the excessive light coupling into the cladding or even leaking into the coating. In addition, since a narrow-linewidth laser with an operating wavelength of 1550 nm is required for the subsequent respiratory monitoring experiments, a balloon-shaped fiber optic sensor with a bending radius of 4.6 mm was selected as the experimental sample for measurement in the subsequent experiments.

### 3.3. Temperature Sensing Characteristics

Because the primary sensing mechanism of the proposed balloon-shaped fiber optic respiration monitoring sensor is through real-time monitoring of the temperature change of the gas, its response to temperature was first investigated. To ensure the linearity of the sensor, the thermostatic stage was gradually increased from 30 °C to 60 °C in 5 °C increments. Each transmission spectrum was recorded after the thermostat stage had reached a stable temperature for 10 min, ensuring the temperature stability of the thermostat.

**Figure 5:**
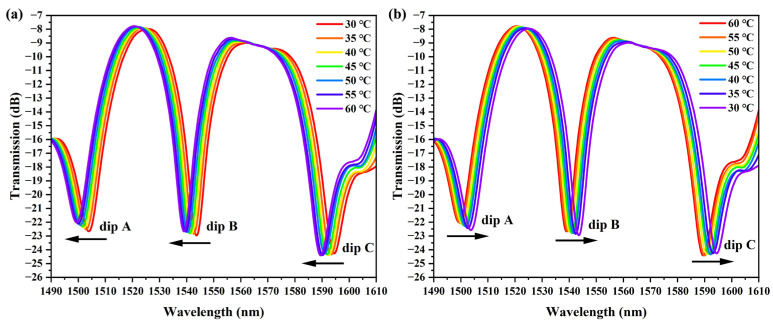
Evolution of transmission spectra with temperature change. (**a**) Increase in temperature; (**b**) decrease in temperature.

The proposed sensor exhibits changes in its transmission spectrum as the temperature of the thermostat increases within a temperature range of 30–60 °C, as shown in Figure 5a. With the increase in temperature, all three interfering dips of the sensor were significantly blue-shifted, mainly due to the negative values of TOC and TEC of the PDMS coated by the SMF, which is by the theoretical derivation of Equation (4). Dip A, with a wavelength position of 1503.92 nm, was blue-shifted by 4.56 nm; dip B, with a wavelength position of 1543.76 nm, was blue-shifted by 4.8 nm, and dip C, with a wavelength position of 1594.40 nm, was blue-shifted by 5.04 nm. To test the temperature repeatability of the balloon-shaped fiber optic sensors, a reverse measurement cycle was performed, and the results are shown in Figure 5b. As the temperature decreases from 60 °C to 30 °C, the evolution of the interference dips of the sensor is opposite to the temperature increase.

**Figure 6:**
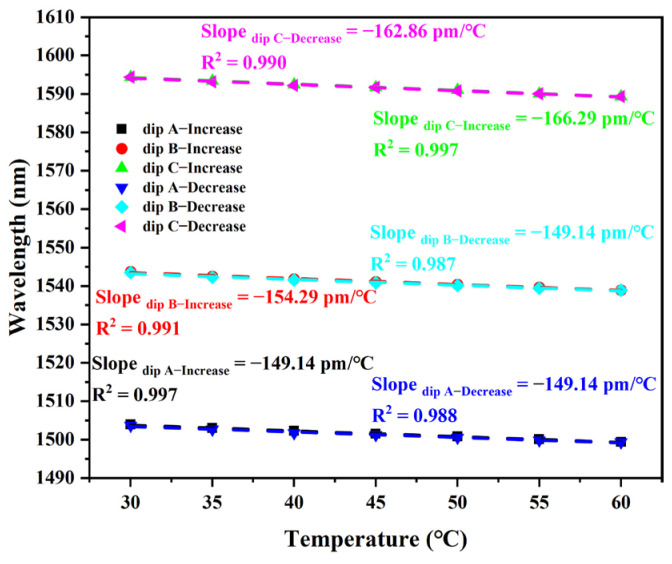
Linear fit curve of wavelength shifts of interference dips with temperature change.

Based on the results of the temperature sensing experiment, it can be seen that the proposed sensor has a significant response to temperature variations. Figure 6 shows the linear fitting curves of the sensor’s dips versus temperature for both increasing and decreasing temperatures. When the temperature increases from 30 °C to 60 °C, the temperature sensitivities of dip A, dip B, and dip C are −149.14 pm/°C, −154.29 pm/°C, and −166.29 pm/°C, respectively. When the temperature of the thermostat was reduced from 60 °C to 30 °C, the temperature sensitivities of dip A, dip B, and dip C were −149.14 pm/°C, −149.14 pm/°C, and 162.86 pm/°C, respectively. According to the linear fitting results, the proposed balloon-shaped fiber optic sensors exhibit good linear regression values (R^2^ ≥ 0.987) for changes in external environmental temperature. Based on the variation in the transmission spectral characteristics with temperature and the determined sensitivity, the maximum error of the experimental results for temperature cycling measurements is 3.39%. Thus, the sensor has good repeatability.

**Figure 7:**
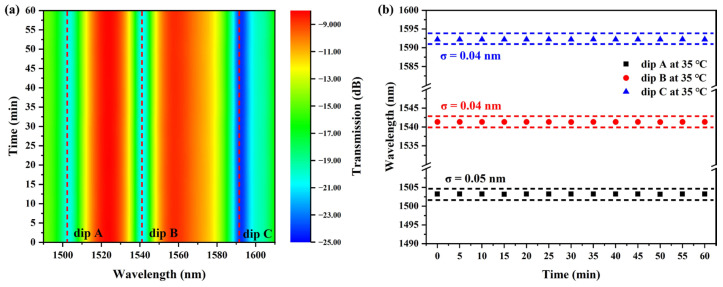
Stability of the sensor at 35 °C. (**a**) Variation in transmission spectrum. (**b**) Variation in interference dips.

To verify the temperature stability of the proposed sensor, the sensor was placed on a thermostat at 35 °C, and the transmission spectrum of the sensor was recorded every 5 min. Figure 7 shows the stability measurement results of the sensor at 35 °C over 1 h. The results indicate that the maximum wavelength shift of the interference dips is approximately ±0.05 nm, demonstrating that the sensor has good time stability.

### 3.4. Temperature Response Time

**Figure 8:**
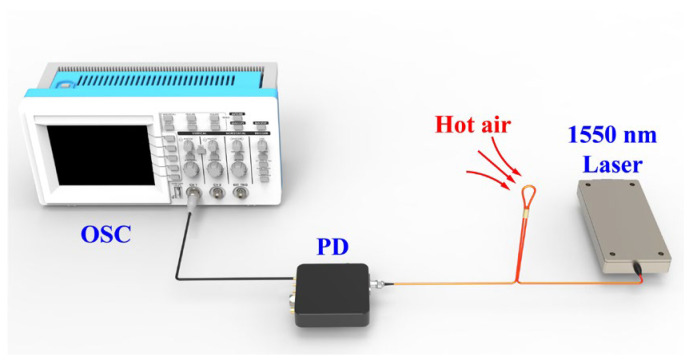
Schematic diagram of the temperature response test setup.

**Figure 9:**
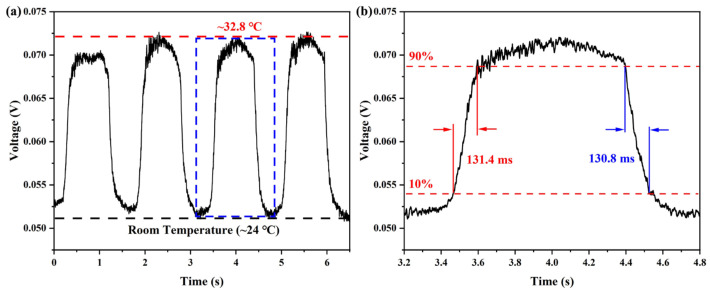
Response time experiments for temperature detection. (**a**) Response time characteristics of the sensor at temperatures ranging from 24 °C to 32.8 °C. (**b**) Localized zoom of the time response characteristic plot to evaluate the response and recovery time of the sensor.

Temperature response time is a crucial factor for the proposed sensor to be effectively applied in respiratory condition monitoring. To measure its temperature response time, the temperature sensor was synchronized with a commercial temperature probe. The test setup is shown in Figure 8; the output signal of the proposed temperature sensor was converted from an optical signal to an electrical signal by a photodetector (PD, Newport, CA, USA, Model 1617-AC) with a bandwidth of 100 kHz and recorded by an oscilloscope (OSC, Tektronix, OR, USA, MDO3104). The light source used was a 1550 nm narrow linewidth laser (RIO Lasers, Santa Clara, CA, USA, ORION 1550 nm Laser Module). First, the blow-in hot air temperature was recorded several times at room temperature (24 °C) using a commercial temperature sensor. The average temperature of the hot air was approximately 32.8 °C. Then the balloon-shaped fiber optic temperature sensor was placed in the same position as the commercial temperature sensor and hot air was regularly blown into the sensor. The experimental results are shown in Figure 9a, where the optical signal varies periodically in response to the rhythm of heat exchange. As shown in Figure 9b, the best response time of the balloon-shaped fiber optic temperature sensor (defined as 10% to 90% of the maximum output power level) was 131.4 ms. Additionally, the sensor has a best recovery time was 130.8 ms.

### 3.5. Wearable Respiratory Masks

**Figure 10:**
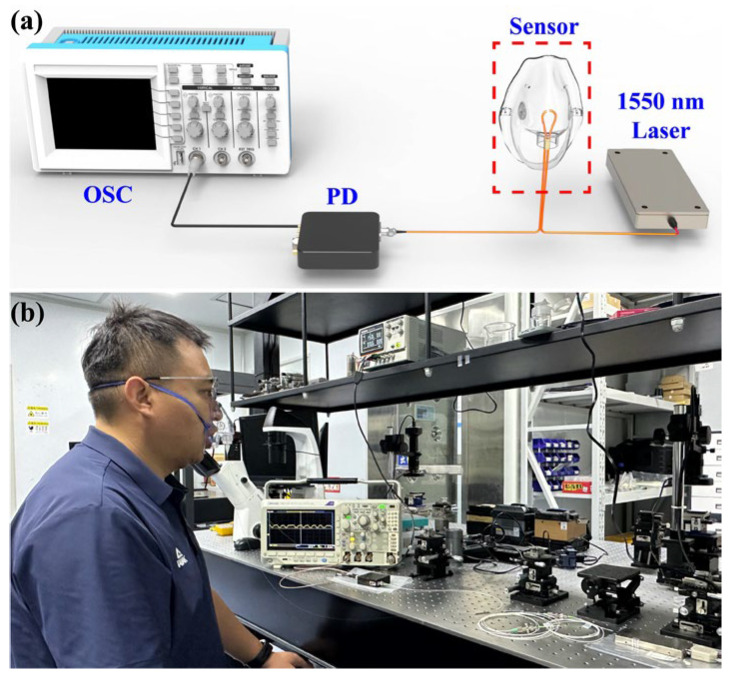
Experimental platform for human respiratory monitoring. (**a**) Schematic diagram of the respiration monitoring sensor. (**b**) Volunteer testing breathing status with the proposed sensor.

**Figure 11:**
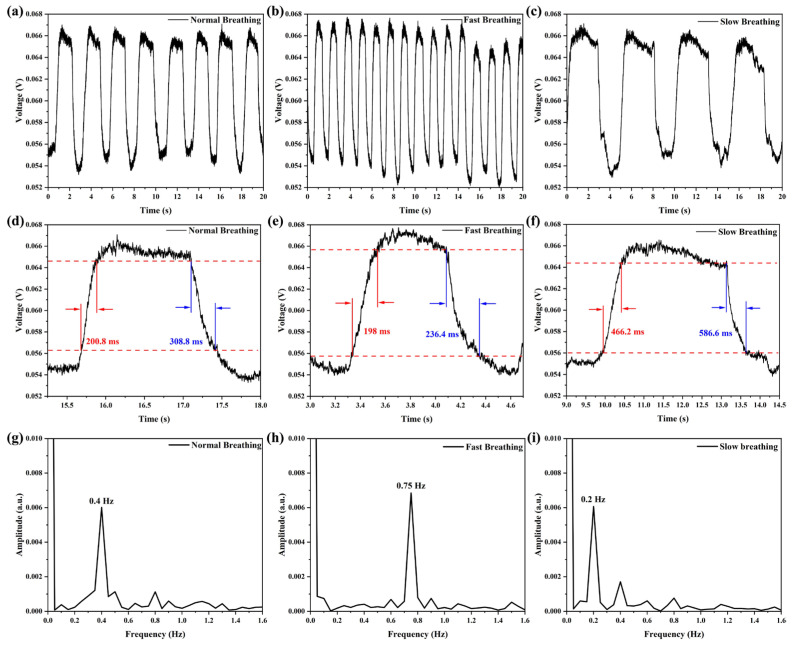
Response characteristics of the sensor under different breathing states. (**a**–**c**) Temporal response characteristics under different breathing states, (**d**–**f**) response time and recovery time under different breathing states, and (**g**–**i**) breathing frequency under different breathing states.

The breathing status monitoring sensor device is shown in Figure 10a. Based on the temperature response of the balloon-shaped fiber optic sensor, this sensor is fixed to the respirator mask for breathing status monitoring experiments. The balloon-shaped fiber optic sensor is connected to the 1550 nm narrow linewidth laser and the PD. The PD converts the transmitted optical signals into electrical signals in real time. These electrical signals are displayed, saved, and analyzed by the OSC. Figure 10b shows a volunteer testing the breathing state with the proposed wearable respiratory sensor. Figure 11 shows the breathing waveforms of the volunteer under three different breathing states. During exhalation, the signal voltage rises sharply with the rapid increase in temperature inside the mask. During inhalation, the signal voltage drops rapidly until it returns to the original signal strength. The sensor monitors a normal breathing rate of 0.4 Hz at rest, a fast breathing rate of 0.75 Hz after exercise, and a slow breathing rate of 0.2 Hz at rest. Moreover, the fastest response and recovery times of the wearable respiratory sensor were 198 ms and 236.4 ms, respectively.

**Figure 12:**
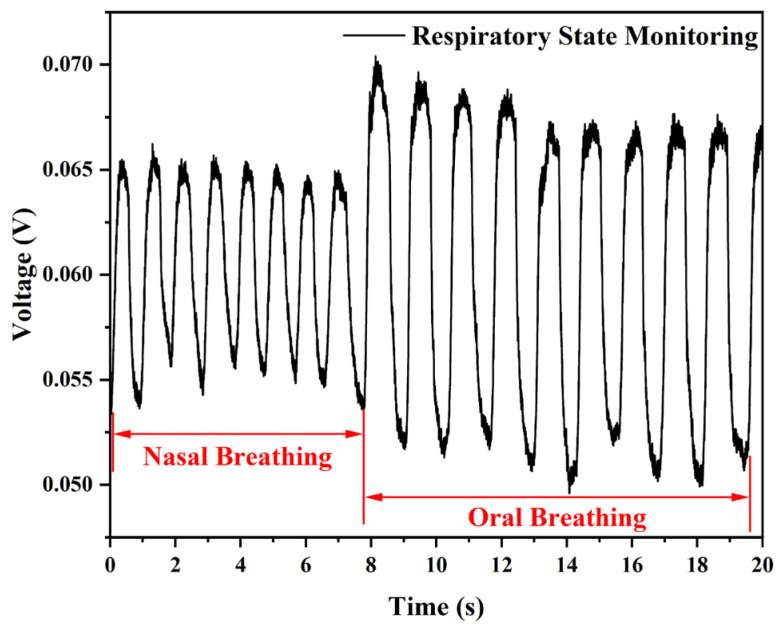
The sensor is monitoring the change from nasal to oral breathing.

The recovery time of the sensor was longer than the response time in the three different breathing states due to the slow rate of temperature change resulting from the relative closure in the respiratory mask. This phenomenon is evident in the deep, slow breathing shown in Figure 11f, where the rate of temperature change in the mask slows down as the inspiratory time increases, resulting in a recovery time of 586.6 ms. When switching to the fast breathing state, the rate of temperature change in the mask accelerates, and the recovery time decreases to 236.4 ms. Additionally, there is a slight difference in the temperature of the exhaled gas, depending on the temperature of the oral cavity and the nose. This temperature difference can serve as a basis for the proposed sensor to monitor the conversion between oral and nasal breathing states, as demonstrated in the experimental results shown in Figure 12. Volunteers initially used nasal fast breathing during exercise and then switched to oral fast breathing, resulting in a significant enhancement of the voltage signal on the OSC. Therefore, the proposed sensor can monitor an athlete’s breathing state in real time during exercise, providing a means to assess the athlete’s physical state.

### 3.6. Performance Comparison

Table 1 shows respiratory sensors for different monitoring parameters. Among them, fiber optic respiratory sensors based on monitoring relative humidity account for the majority. However, to improve the humidity sensitivity and response time of the sensor, complex structural processing of the fiber optic is required, which also results in lower robustness and higher preparation costs for the sensor. The proposed sensor avoids complex fabrication processes, enabling the low-cost manufacture of a compact structure while exhibiting response and recovery times comparable to those of most sensor types.

## 4. Conclusions

In summary, a fiber optic respiratory monitoring sensor based on a balloon-shaped structure is proposed. The sensor bends the PDMS-coated SMF into a balloon-shaped structure with a radius of 4.6 mm, effectively exciting higher-order modes to interfere with the core mode, forming a fiber optic MZI. The sensor monitors breathing status and frequency by detecting the temperature changes generated during a person’s breathing. The experimental results show that within a temperature range of 30–60 °C, the temperature sensitivity is −166.29 pm/°C, and the response time and recovery time to temperature are 131.4 ms and 130.8 ms, respectively. Finally, the sensor was attached to a respiratory mask to monitor various breathing states (normal breathing, fast breathing, slow breathing, and transitions between oral and nasal breathing states) and breathing frequency. With the advantages of simple fabrication, high sensitivity, high stability, and fast response speed, the sensor has a broad application prospect in the fields of public health management and competitive sports.

## Figures and Tables

**Table 1:** Comparison of different fiber sensors for respiratory monitoring.

Sensor Type	Sensitivity	Response Time	Recovery Time	Reference
Side-Polished Optical Fiber-MoS_2_	-	850 ms	850 ms	[17]
Eccentric Fiber Bragg Grating	-	92 ms	100 ms	[9]
Fiber SPR based on SF-LiBr film	−6.5 nm/%RH	135 ms	150 ms	[12]
Micro-Tapered Long-Period Grating	0.048 dB/%RH	237 ms	299 ms	[18]
Side-Polished SMF-HCF-SMF-PDMS	4.2 nm/°C	16 ms	176 ms	[20]
**Balloon-Shaped Fiber Optic** **-PDMS**	**166 pm/°C**	**131.4 ms**	**130.8 ms**	**This work**

## Data Availability

The data will be available upon request.
